# Treating Depression in Dementia-patients with transcranial-pulse stimulation: an observational study

**DOI:** 10.1192/j.eurpsy.2026.10717

**Published:** 2026-07-31

**Authors:** A. R. Günes, M. Rahimi, P. Wisniowski, M. Beglau, M. Köhne, C. Schmidt-Kraepelin, U. Sprick

**Affiliations:** 1 Center of Neurostimulation; 2 Alexius/ Josef Clinic, Neuss, Germany

## Abstract

**Introduction:**

Depression plays a significant role in Alzheimer’s-type dementia(AD) and affects approximately one in five patients^1^ (Asmer MS *et al.* J Clin Psychiatry. 2018 Jul 31;79). On the other hand, depression is a risk factor for the development of dementia. Despite years of research and the discovery of numerous pathophysiological factors in their mutual influence, there is still no sufficiently reliable therapeutic approach^2^ (Lyketsos CG *et al.* Alzheimers Dement.2011;7:5). As a non-invasive brain stimulation method, transcranial pulse stimulation (TPS) is a novel procedure based on shock-waves and whose effects on cognition in AD patients show promising results in current studies^3^(Günes AR *et al.* Eur.J.N.2024;1468-1331), furthermore beneficial influence on affective symptoms were reported^4^ (Sprick U *et al.* Brain Stimul.2025;439).

**Objectives:**

In our observational study, we investigated the effects and safetiness of TPS on depressive symptoms in 100 patients who met the criteria for AD.

**Methods:**

Each of the 100 patients (mean age: 73,5 years; gender ratio: 5:7 (m:f )) received a total of 6 treatments within 14 days (each session with 6000 pulses, energy level 0.25 mJ/mm² and frequency 4 Hz). On the basis of individual MRI scans neuro-navigation was used to apply pulses bilaterally in the frontal (including the dorsolateral prefrontal cortex) and parietal lobe as well as the precuneus area (Image 1). Written consent and the approval of an ethics committee were obtained for all patients. Before, during and immediately after the TPS intervention, the antidepressant medication of all patients remained unchanged. To assess the intensity of depressive symptoms the Beck Depression Inventory II (BDI-II) was used before the first and after the last treatment session.

**Results:**

36 patients had a score above 5 points in BDI-II which represents the lower threshold with an average of 18,1 points within this group, which corresponds to a mild depression. The BDI-II total score in the pre-post comparison showed a reduction in the mean value from 18,1 to 5,9 points indicating a score almost below the lower threshold (Image 2). 26 patients reported temporary mild side effects such as fatigue or a local feeling of pressure in the treated areas. There were no serious adverse effects detected in any patient.

**Image 1:**
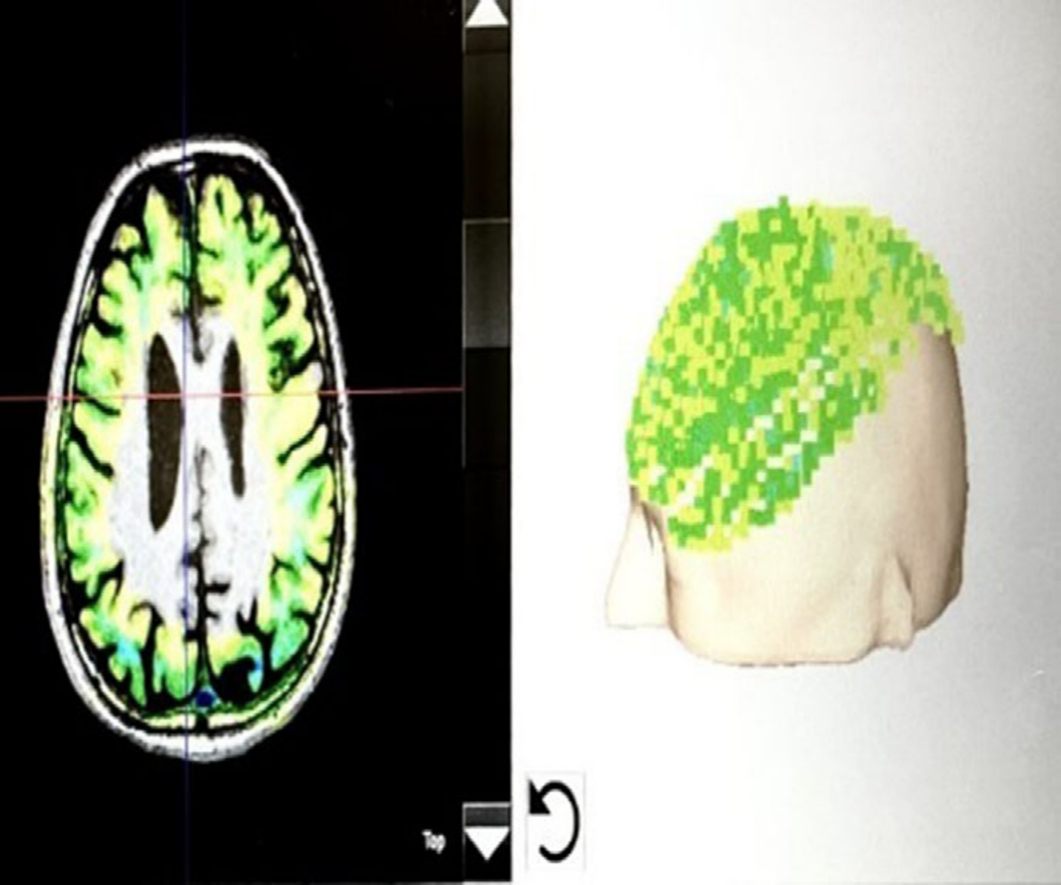
Image 1: Long description.

**Image 2:**
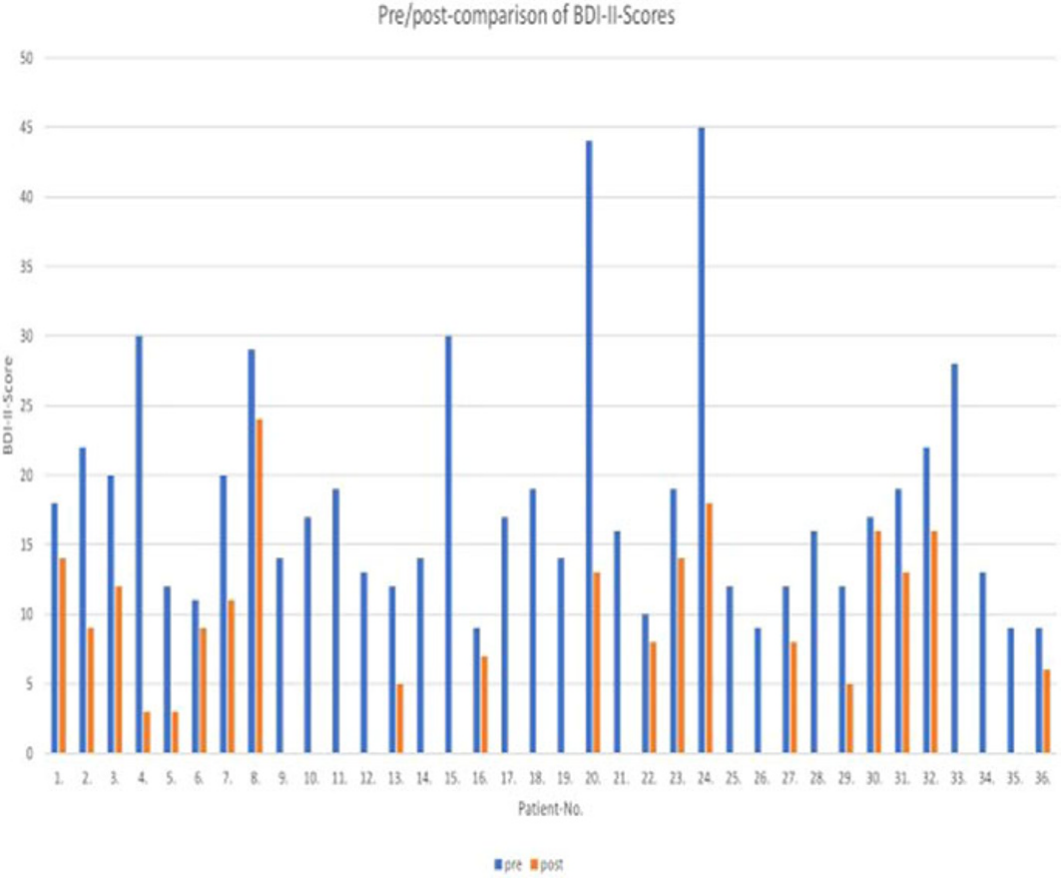
Image 2: Long description.

**Conclusions:**

To our knowledge, this trial is the largest study to systematically investigate the effects of TPS on depressive symptoms in AD patients so far. The positive impact on depressive symptoms may be based in part on stimulation of the dorsolateral prefrontal cortex, which is a well established target region in the treatment of major depressive disorder using transcranial magnetic stimulation. TPS seems to be a safe approach to treat depressive symptoms in patients with AD. Further studies with a larger study population are necessary to confirm the safety and effectiveness of this novel method.

**Disclosure of Interest:**

None Declared

## Long descriptions

### Image 1: Long description

Panel one on the left is an axial M R I scan of a human brain. At the center are the dark, butterfly-shaped lateral ventricles. Surrounding them is the white matter, shown in bright white. The outermost layer is the cerebral cortex, displaying intricate folds of gyri and sulci highlighted with green and yellow tints. A red horizontal line and a blue vertical line intersect at the center of the scan. Panel two on the right is a three-dimensional profile view of a human head facing left. The top and frontal regions of the skull are covered by a dense, pixelated map of small green and yellow squares, representing data localized to the scalp or cortical surface. A small circular arrow icon for rotation is located at the bottom left of this panel.

Navigate back to Image 1:.

### Image 2: Long description

A bar chart titled Pre forward slash post dash comparison of B D I dash I I dash Scores. The Y-axis is labeled B D I dash I I dash Score with a scale from 0 to 50 in increments of 5. The X-axis is labeled Patient dash No. and lists 36 individual patients. For each patient, there are two bars: a blue bar representing pre-treatment and an orange bar representing post-treatment.

* General Trend: In almost every case, the orange post-treatment bar is lower than the blue pre-treatment bar, indicating a reduction in depression scores. Several patients (9, 10, 11, 12, 14, 15, 17, 18, 19, 21, 22, 25, 26, 28, 33, 34, and 35) have no visible orange bar, suggesting a score of zero or near-zero post-treatment.

* Peak Values: Patient 24 has the highest pre-score at 45, which drops to 18 post-treatment. Patient 20 has the second-highest pre-score at 44, dropping to 13.

* Minimal Change: Patient 8 shows a high pre-score of 29 and a relatively high post-score of 24. Patient 30 shows a pre-score of 17 and a post-score of 16.

* Low Baseline: Patients 35 and 36 have the lowest pre-scores, both at 9.

Navigate back to Image 2:.

